# A review of key strategies to address the shortage of analgesics and sedatives in pediatric intensive care

**DOI:** 10.3389/fped.2022.895541

**Published:** 2022-08-30

**Authors:** Roberta Esteves Vieira de Castro, Miguel Rodríguez-Rubio, Maria Clara de Magalhães-Barbosa, Arnaldo Prata-Barbosa, Jaimee Holbrook, Pradip Kamat, Anne Stormorken

**Affiliations:** ^1^Pediatric Intensive Care Unit, Rio de Janeiro State University, Rio de Janeiro, RJ, Brazil; ^2^Department of Pediatrics, D'Or Institute for Research and Education, Rio de Janeiro, RJ, Brazil; ^3^Department of Pediatrics, Souza Marques School of Medicine, Rio de Janeiro, RJ, Brazil; ^4^Pediatric Intensive Care Department, Hospital Universitario La Paz, Madrid, Spain; ^5^Department of Pediatrics, Facultad de Medicina, Universidad Autónoma de Madrid, Madrid, Spain; ^6^Department of Pediatrics, University of Chicago Medicine, Chicago, IL, United States; ^7^Department of Pediatrics, Emory University School of Medicine, Atlanta, GA, United States; ^8^Department of Pediatrics, Case Western Reserve University School of Medicine, Cleveland, OH, United States

**Keywords:** pediatrics, COVID-19, sedation, analgesia, delirium

## Abstract

**Importance:**

Targeted analgosedation is a challenge in critically ill children, and this challenge becomes even more significant with drug shortages.

**Observations:**

Published guidelines inform the provision of analgosedation in critically ill children. This review provides insights into general approaches using these guidelines during drug shortages in Pediatric Intensive Care Units as well as strategies to optimize both pharmacological and non-pharmacological approaches in these situations.

**Conclusions and relevance:**

Considering that drug shortages are a recurrent worldwide problem, this review may guide managing these drugs in critically ill children in situations of scarcity, such as in pandemics or disasters.

## Introduction

In 1955, the pioneer dedicated Pediatric Intensive Care Unit (PICU) was established in Europe, and pediatric critical care medicine has only been accepted as a distinct specialty since 1981 (1). Many pediatric critically ill patients in these units require life-saving measures, including mechanical ventilation (MV), continuous renal replacement therapy, and extracorporeal membrane oxygenation therapy. These interventions are associated with a protracted PICU stay and the need for analgosedation (2).

Unfortunately, several essential analgesic and sedative agents have been depleted globally primarily due to increased needs and the disruption of manufacturing and supply chains (3). In 2016, the World Health Organization proactively took a stand about the lack of essential medicines, reported in low, middle, and high-income countries. The scarcity of drugs poses risks to the patient's health due to non-treatment, undertreatment, and failure to find suitable alternatives (4).

This review aims to: (1) provide the reader with information regarding drug supply chain issues; (2) highlight the levels of service for dispensing medicines: conventional, contingency and crisis, emphasizing the role of pharmacists in the rationing of them; (3) address the relevance of appropriate management of analgesia and sedation in the PICU; and (4) present dose optimization strategies regarding analgo-sedative choices in addition to considering different approaches to preventing medication overuse.

## Drug shortages: A global issue

Numerous definitions for drug shortages have been widely used, as shown in Table 1. Unfortunately, there is a lack of a standardized definition. Moreover, the low and middle-income countries have the absence of an official description (5).

**Table 1:** Definitions of drug shortages according to different institutions.

**Institution**	**Definition**
ASHP and UUDIS	“A supply issue that affects how the pharmacy prepares or dispenses a drug product or influences patient care when prescribers must use an alternate agent.”
EFPIA	“A crisis situation caused by any ability of any MAH to supply a medicine with a specific API to market over an extended period of time resulting in the unavailability of this medication for patients.”
FIP	“A drug supply issue requiring a change. It impacts patient care and requires the use of alternative agents.”
Health Canada	“When a manufacturer/importer anticipates that they cannot supply a drug to meet projected demand.”
ISPE	“A situation in which total supply of an approved medicine is inadequate to meet the current projected demand at the user level.”
US FDA (three definitions)	1. “A period of time when the demand or projected demand for drug exceeds the supply of drug.” 2. “When demands exceeds supply at any point in the supply chain may ultimately create a “stock-out” at the point of appropriate service delivery to the patient if the cause of shortage cannot be resolved in a timely manner relative to the clinical needs of the patients.” 3. “A situation in which the total supply of all clinically interchangeable versions of an FDA regulated drug product is inadequate to meet the projected demand at the user level.”

API, active pharmaceutical ingredients; ASHP, American Society of Hospital Pharmacists; EFPIA, European Federation of Pharmaceutical Industries and Associations; FIP, International Pharmaceutical Federation; ISPE, International Society of Pharmaceutical Engineering; MAH, Market Authorization Holder; US FDA, Food and Drug Administration (United States); UUDIS, University of Utah Drug Information Service (5).

Drug shortages are recognized as a global issue (4) and are usually due to several factors. Drug shortage affects high, middle, and low-income countries. In high-income countries, it has more attention when compared to other regions of the globe. The supply chain for delivering raw materials to patient use is complex and involves multiple entities, including manufacturers, group purchasing associations, wholesalers, and healthcare systems. On the manufacturing side, drug shortages occur due to lack of raw materials, regulatory problems, manufacturing interruptions, voluntary and involuntary recalls, promotion reduction (such as patent expiration or generic drug profitability), or manufacturer consolidations. Moreover, drug shortages also occur due to improper stocking practices, changes in clinical practice (resulting in increased demand), and even supply chain disruption due to natural disasters. Otherwise, low-middle income countries have some novel reasons for drug shortage, comprising licensing of manufacturers/products, shortage of raw material for a local producer, drug smuggling, and lodging tax government practices. These countries have insufficient research and lack policies to deal with this problem (5–7).

During a major disaster, such as a pandemic or war, for example, it is necessary to forecast and manage the shortage of drugs essential to critical care from global, national, regional, and institutional perspectives. Drug shortages can be expected to coincide with an interruption of other necessary resources such as personnel, availability of personal protective equipment, and medical devices. Supplies for drug preparation and administration can also be scarce (3).

Burry et al. summarized the main strategies that stakeholders must consider for future steps during a global disaster from the worldwide level to the institutional approach:

- Global: Proactively plan for shortages *via* substitution and conservation strategies; establish transnational networks with national and regional sharing arrangements; develop recommendations on essential supplies.
- National and Regional Manufacturing: create usage prediction models; couple with inventory management; engage manufacturers; improve pharmaceutical processes; eliminate redundant critical production steps; recommendations on essential supplies; collaborative dashboards; sharing arrangements; decentralize production to multiple sites. Supply disruption is exacerbated if there are limited manufacturing capacity, market concentration, or just-in-time inventory practices that result in minimal product inventory on hand at any given time. However, the Food and Drug Administration (FDA) cannot prevent manufacturing concentration, require redundancy of that capability, require a company to manufacture a drug, maintain a certain level of inventory of the drug, or reverse a business decision to stop manufacturing. Manufacturers may consider opportunities to increase redundant manufacturing capacity, maintain idle capacity, or increase inventory levels to reduce shortage risks, and other stakeholders can explore how to encourage such practices.
- Institutional: Balance drug inventory; identify drugs at risk of shortage; develop drug conservation guidelines; rotate stock; identify therapeutic alternatives (3, 8).

Given this situation, the role of pharmacists in alleviating the current crisis and future challenges is a central one. Notably, among the pharmacist's actions is advocacy for implementing the *Interagency Drug Shortage Task Force* recommendations by participating in dedicated drug shortage task forces or rationing committees to guide management strategies and keeping informed regarding drug shortages (9).

Finally, Ammar et al. suggest that drug escalation capacity and response be measured based on three levels: **conventional care**, **contingency care**, and **crisis care**. Contingency care comprises all the practices that may be outside usual care notwithstanding they attempt to keep traditional care. On the other hand, crisis care approaches are outside of standard of care, however, provide the best feasible care when resources are severely limited (10).

Children have unique illnesses and are at a singular developmental stage that may need specific medications for which there may not be therapeutic alternatives. Additionally, the evidence supporting **the use of substitutes may be limited in pediatric patients and may raise concerns for adverse events**. Therefore, a comprehensive and multidisciplinary approach is necessary to ensure that drug shortages do not lead to unfavorable patient outcomes (11).

## The challenges of analgesia and sedation in pediatrics

Alleviating pain and anxiety in critically ill children may be quite challenging. Patient admission and daily care processes within PICUs can be frightening and painful for pediatric patients and their families. Pain can result from the underlying disease or trauma and can be exacerbated by anxiety and emotional stress, two common elements of the PICU stay. The condition in which children find themselves in this environment, surrounded by strange people and machines, separated from their parents, in a hostile, noisy, and bright place most of the time, leading to the interruption of the circadian cycle, causes more anxiety and vulnerability to pain. Pain may also result from diagnostic and therapeutic interventions to which patients are submitted during the hospitalization period. In intensive care, children, and newborns (NB) are often subjected to numerous potentially uncomfortable or painful procedures, such as arterial and venous punctures, thoracic drainage, and endotracheal intubation. These therapeutic interventions place an enormous burden on these patients, affecting the successful performance of these procedures and the patient's recovery (12–16).

An effective analgesia approach facilitates invasive procedures or interaction with invasive equipment such as MV and enhances rehabilitation of the critically ill patient (12). Accurate assessment of pain and comfort using validated scales with targeted, measured goals is central to excellent clinical management (13).

Adequate analgesia and sedation minimize the stress response and improve clinical and psychological outcomes. When inadequate, negative outcomes include undertreated pain or persistent agitation leading to accidental removal of invasive devices. On the other hand, oversedation results in prolonged PICU and hospital stay, prolonged MV, and the development of tolerance, physical dependence, iatrogenic withdrawal syndrome (IWS), and delirium. Accurate assessment of pain, distress, IWS, and delirium in critically ill children can be challenging as these conditions often overlap. The use of validated assessment scales as outlined in the recent PANDEM guidelines facilitates assessment guiding proper care and mitigating their development (12, 17).

The *2018 Clinical Practice Guidelines for the Prevention and Management of Pain, Agitation/Sedation, Delirium, Immobility, and Sleep Disruption in Adult Patients in the ICU* (PADIS Guidelines) have recommended the approach to sedation as “analgesia first” (before sedation) or analgesia-based sedation, which implies that an analgesic (usually an opioid) is used before a sedative to achieve the desired sedative goal. Institutions should have protocols that include periodic evaluations of pain and sedation using validated tools and provide clear guidance on the choice and dose of the drug, ensuring that pain treatment is a priority over the administration of sedatives (18). Recently, Smith et al. developed Society of Critical Care Medicine (SCCM) clinical practice guidelines for critically ill pediatric patients, including pain, sedation/agitation, iatrogenic withdrawal, neuromuscular block, delirium, PICU environment, and early mobility. Key areas included the need for routine monitoring of pain, agitation, withdrawal, and delirium using validated tools in children; improved use of protocol sedation and analgesia, and recognition of the importance of non-pharmacological interventions to improve patient comfort and provision of comprehensive care (17).

The SCCM developed a multicomponent and evidence-based six-step strategy to liberate patients from the ICU. This approach, called “the ABCDEF bundle,” represents **A**: Assess, prevent, and manage pain; **B**: Both Spontaneous Awakening Trials (SATs) and Spontaneous Breathing Trials (SBTs); **C**: Choice of sedation; **D**: Delirium assessment, prevention, and management; **E**: Early mobility and exercise; and **F**: Family engagement and empowerment. The conceptualization of the proposed “ICU Liberation” achieved notable recognition in adult critical care research and has become prominent in PICU. In a recent survey of 161 PICUs in 18 countries, Ista et al. observed that, unfortunately, the A-F bundle items have been adopted with substantial variability internationally (19).

We should emphasize that providing analgosedation is not restricted to the PICU environment. Currently, with the exponential growth in the number of procedures in children and adolescents experience outside the operating room, there has been a greater need for awareness and guidance on procedural sedation by professionals who are not anesthesiologists, including emergency departments, wards, outpatient clinics, imaging centers, and dental offices (20, 21).

Regarding drug shortages, in the specific case of analgosedation, one of the recommended approaches to conserving intravenous analgesic supplies during contingency care is to implement protocols where clinicians would initially use intermittent analgesic boluses before patients transition to continuous infusion. In addition, enteral delivery of opioids and analgesics may help to conserve the supply of intravenous (IV) agents. However, this strategy should be limited to patients with adequate gastrointestinal motility and function. Other situations can also be considered. For example, ketamine has analgesic properties and may spare the use of IV opioids. Furthermore, it is not known to cause significant respiratory depression at moderate doses, and this is advantageous when trying to transition the child off MV (10).

With regard to benzodiazepines, they can also be administered in intermittent doses or as a continuous infusion to obtain mild sedation. However, they should only be considered as first-line sedatives in contingency care settings. When used, IV lorazepam, midazolam, or diazepam in scheduled doses or as needed, can help to conserve drug stocks in the scenario of ongoing shortages. This approach can limit overall sedative exposure while still providing appropriate light sedation, preserving the need for high doses and continuous infusions known to be associated with accumulation. However, if this is not sufficient for adequate sedation, continuous infusion of benzodiazepines can be started. Still intermittent doses may be reconsidered again when continuous infusion is no longer needed and transition from continuous infusion to a less aggressive dose is appropriate (10).

## Dose optimization strategies

Analgo-sedative regimen selection should take into consideration patient-specific risk factors, targeted level of sedation, anticipated duration, analgesic needs, physician familiarity, and institutional formulary availability. The following strategies that ensure comfort and optimize dosing of analgesics and sedatives. Applying these strategies can be challenging for teams unfamiliar with these measures, particularly when human resources are scarce, and family presence is restricted.

### Set a goal and reassess regularly and as patient condition changes

It is highly relevant for the interdisciplinary team to actively participate and discuss the goals of analgesia and sedation when necessary. When determining a sedative and analgesic regimen for a critically ill patient, the first step is to choose the desired degree/depth of sedation.

Moreover, the “Pediatric Brain Roadmap” contributes like a script to disseminate delirium assessment results and crucial information to guide delirium management discussion during interdisciplinary rounds. Its components are pain assessment, target, and actual LOC, delirium assessment, and sedative/analgesic/antipsychotic medications previously received:

- Where is the patient going? → Sedation targets and therapy goals.
- Where is the patient now? → Actual level of consciousness (RASS)/Delirium assessment/Pain assessment.
- How did they get there? → Shock, hypoxia, fever, drug exposure (18, 22).

### Whenever possible, target a patient with RASS 0 (alert and calm)

There are few current indications for continuous deep sedation. These include the treatment of intracranial hypertension, severe respiratory failure, refractory status epilepticus, and prevention of consciousness in patients treated with neuromuscular blocking agents (18, 22).

### Consider non-pharmacological strategies for analgesia and sedation

Non-pharmacological interventions can reduce the total requirement and associated side effects of sedation and analgesia medications and have been recommended by international sedation guidelines. In addition to addressing risk factors, these strategies include daily screening for delirium; environmental orientation; maintaining normal hydration; regulation of bladder and bowel function; early establishment of normal diet; correction of metabolic disorders; cardiorespiratory optimization; early identification of infection; effective treatment of pain; daily mobilization; avoidance of antipsychotic drugs, benzodiazepines, and anticholinergics; sleep promotion; light and noise reduction; early removal of invasive devices; avoidance of physical restraints; attention to the parameters and modes of ventilation; cluster care (18, 22–24).

### Use the “analgesia first” or “analgesia-based sedation” approach

Consider pain assessment and treatment with opioid-sparing measures using a multimodal analgesia strategy, including non-opioid analgesics such as acetaminophen, dipyrone (metamizole), nefopam, ketamine, lidocaine, neuropathic agents, and NSAIDs (18).

### Patient-specific drug therapy

Once the depth of sedation is chosen, it is essential to focus on selecting specific sedatives and pain relievers. In recent years, numerous studies have shown that benzodiazepines are independently associated with the incidence of delirium. Therefore, benzodiazepines should not be used as first-line sedatives in critically ill children. In the last decade, the use of alpha agonists such as dexmedetomidine has increased in PICUs. It may shorten the duration of MV and reduce the need for opioids and the incidence of delirium. In addition, attention should be paid to the individual characteristics of each patient. Care should be taken concerning obese patients and those with organ dysfunction and arrhythmias (18, 24, 25).

### Medication rotation

Some authors suggest establishing a sedation rotation regime based on the hypothesis that replacing sedative and analgesic drugs targeting different receptors for shorter periods may decrease the incidence of tolerance and IWS (26).

### A-F bundle

This bundle promotes fast recovery and ICU liberation, with satisfactory evidence in adults and children.

- *[A] Assessment and management of pain*.
- *[B] Both awakening and breathing trials*.
- *[C] Choosing the optimal sedative (avoiding benzodiazepines when possible) and titrating to the lightest sedation level possible*.
- *[D] Delirium assessment and management*.
- *[E] Early mobility and exercise*.
- *[F] Family engagement and empowerment when possible* (27–31).

The inclusion of the letter R (respiratory-drive-control“ “ABCDEF”R”) was suggested by Chanques et al. and should be considered to prioritize the management of factors related to the MV and the respiratory unit, avoiding the unnecessary use of medications that can delay ventilator release and worsen other patient outcomes (32).

In Table 2, we summarize the pharmacological options for providing analgesia and sedation in conditions of scarcity.

**Table 2:** Alternatives for pediatric analgesics and sedatives according to the desired sedation level.

**Alternatives for pediatric analgesics**			
**First-line analgesics—Conventional care**			
	**Light sedation**	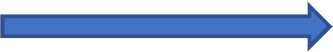	**Deep sedation**
Fentanyl or morphine AN			Fentanyl or morphine CI
Scheduled acetaminophen, PO, or IV			Scheduled acetaminophen, PO, or IV
Scheduled gabapentin or pregabalin (in case of neuropathic pain)			Scheduled gabapentin or pregabalin (in case of neuropathic pain)
**Second-line analgesics—Conventional care**			
	**Light sedation**	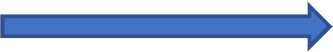	**Deep sedation**
Ketamine AN			Ketamine CI^▾^
Hydromorphone AN			Hydromorphone CI
Oxycodone immediate release AN			Scheduled oxycodone immediate release
**Third-line analgesics—Contingency care**			
	**Light sedation**	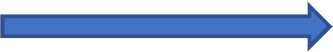	**Deep sedation**
Remifentanil CI ^▴^			Scheduled methadone, PO, or IV
**Fourth-line analgesics—Crisis care**			
	**Light sedation**	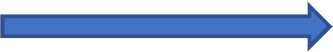	**Deep sedation**
Lidocaine IV			Sufentanil CI
Nefopam PO^|^				
**ALTERNATIVES FOR PEDIATRIC SEDATIVES**			
**First-line sedatives—Conventional care**			
	**Light sedation**	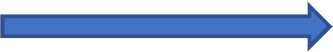	**Deep sedation**
Dexmedetomidine CI^▾^			Ketamine CI^▾^
Ketamine *bolus* IV			Propofol CI^□^
**Second-line sedatives—Conventional care**			
	**Light sedation**	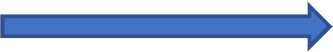	**Deep sedation**
Clonidine PO scheduled every 6 h			Clonidine CI
Midazolam *bolus* IV			Midazolam CI
**Third-line sedatives—Contingency care**			
	**Light sedation**	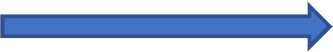	**Deep sedation**
Lorazepam PO or IV scheduled every 4–6 h			Lorazepam CI
Diazepam PO AN			Diazepam PO or IV scheduled every 6–8 h
**Fourth-line sedatives—Crisis care**			
	**Light sedation**	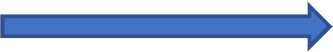	**Deep sedation**
Phenobarbital PO or IV scheduled every 6–8 h			Thiopental CI
Hydroxyzine PO scheduled every 8 h			
Clonazepam PO AN or scheduled			
Atypical antipsychotics^**#**^ (risperidone, quetiapine, and olanzapine) PO AN			
Typical antipsychotics^**#**^ (haloperidol, chlorpromazine, or levopromazine) PO or IV AN			
Chlormethiazole PO AN			

AN, as needed; CI, continuous infusion; IV, intravenous line; PO, orally. ^**▴**^ Remifentanil has single pharmacokinetic and pharmacodynamic profiles. Unfortunately, it is expensive compared to other conventional opioids (33). Current literature has demonstrated that an infusion of lidocaine effectively treats acute perioperative pain and various circumstances of chronic pain in pediatrics, particularly pain refractory to conventional regimens (34, 35). ^|^ Data on nefopam use in children are lacking. However, it is mentioned in the 2018 PADIS Guidelines as an opioid-sparing pharmacological option for pain management (17, 36). ^▾^ As in adults, the use of benzodiazepines in pediatric intensive care is associated with an increased risk of delirium (can be up to four times higher than in children who do not receive them). Therefore, the early addition of dexmedetomidine or ketamine infusion may reduce or even prevent the regular use of benzodiazepines and/or opioids (26, 37). ^□^ According to Koriyama et al., propofol infusions in critically ill children appear to be safe by limiting doses to 4 mg/kg/h and for <24 h; however, adequate follow-up for adverse effects has not yet been carried out due to a lack of solid evidence. Studies show that higher doses and for longer periods are associated with propofol infusion syndrome (38). ^**#**^ In general, typical antipsychotics mainly trigger extrapyramidal syndrome (hyperpyrexia, dystonias, akathisia, Parkinsonism) and hyperprolactinemia. Atypical ones can lead to weight gain and metabolic disorders. Other side effects include malignant hyperthermia, hypotension, laryngospasm, lipid changes, glucose disturbances, and anticholinergic effects. Sedation, increased appetite, and weight gain are more commonly observed with the use of olanzapine (39–44). All antipsychotics carry a risk for QT interval prolongation, with the possibility of torsades de pointes. Risk factors for torsades de pointes include inherent risk of the drug, higher doses, rapid upward titration, rapid IV infusion, female gender, electrolyte disturbance, bradycardia, concomitant QT-prolonging drugs, ion-channel polymorphisms, and patients with congenital long QT syndrome caused by ion channel mutations (45, 46). Moreover, the use of the antipsychotic chlorpromazine in pediatric patients causes numerous drug interactions, ineffectiveness, inappropriate doses, and side effects (47). → For procedural sedation, nitrous oxide is a practical adjunct widely used in dental procedures. It has effective anxiolytic, amnestic, and analgesic, with few side effects associated with its use. Some authors highly recommend its application as part of the sedative arsenal for minor procedures (10, 24, 26, 38, 48–53).

## Conclusion

Care of critically ill children during conditions of scarcity of analgesic and/or sedative drugs has presented numerous challenges globally. Effective approaches to managing drug shortages, implementing evidence-based guidelines for evaluating pain and delirium, and understanding alternative pharmacological and non-pharmacological options for analgesia and sedation will ensure safe and effective management of pain and delirium in the setting of limited resources or future disasters.

## Author contributions

RC conceptualized and wrote the first draft of the review. MR-R, MM-B, AP-B, and JH made substantial contributions to the conception, design, literature data, and content of the tables. PK and AS drafted the article and revised it critically for important intellectual content. All authors approved the final version to be published.

## Conflict of interest

The authors declare that the research was conducted in the absence of any commercial or financial relationships that could be construed as a potential conflict of interest.

## Publisher's note

All claims expressed in this article are solely those of the authors and do not necessarily represent those of their affiliated organizations, or those of the publisher, the editors and the reviewers. Any product that may be evaluated in this article, or claim that may be made by its manufacturer, is not guaranteed or endorsed by the publisher.
